# The analgesic effects of buprenorphine (Vetergesic or Simbadol) in cats undergoing dental extractions: A randomized, blinded, clinical trial

**DOI:** 10.1371/journal.pone.0230079

**Published:** 2020-03-06

**Authors:** Ryota Watanabe, Josée Marcoux, Marina C. Evangelista, Yvan Dumais, Paulo V. Steagall

**Affiliations:** 1 Département de Sciences Cliniques, Faculté de Médecine Vétérinaire, Université de Montréal, St-Hyacinthe, Canada; 2 Centre Hospitalier Universitaire Vétérinaire (CHUV), Faculté de Médecine Vétérinaire, Université de Montréal, St-Hyacinthe, Canada; University of Arizona College of Medicine, UNITED STATES

## Abstract

This study aimed to evaluate the analgesic efficacy of two dosage regimens using two different concentrations of buprenorphine in cats undergoing dental extractions. Twenty-three cats with oral disease (8.2 ± 2.2 years old; 4.9 ± 0.9 kg) were included in a prospective, blinded, randomized clinical trial. Cats randomly received either Simbadol (1.8 mg/mL; 0.24 mg/kg, subcutaneously, every 24h: SG, n = 11) or Vetergesic (0.3 mg/mL; 0.02 mg/kg, intramuscularly, every 8h: VG, n = 12) throughout the study. They were admitted at day 0, underwent oral examination/radiographs/treatment under general anesthesia (buprenorphine-propofol-isoflurane-meloxicam-local anesthetic blocks) at day 1 and discharged at day 4. Sedation and pain were scored using the dynamic interactive visual analog scale (day 1) and the Glasgow Composite Measure Pain Scale-Feline (CMPS-F; up to postoperative 8 hours at day 1, 8 am, 4 pm and midnight at days 2 and 3, and 8 am at day 4), respectively. Rescue analgesia was administered with hydromorphone (0.05 mg/kg intravenously on day 1 or 0.1 mg/kg intramuscularly after day 2) when CMPS-F ≥ 5. Resentment defined as any type of escape behavior associated with aversion to drug administration was recorded. Sedation and pain scores, the prevalence of rescue analgesia and resentment during drug administration were analyzed using linear mixed models and Fisher’s exact test, respectively (*p* < 0.05). Pain and sedation scores were not significantly different between groups. Sedation scores were significantly higher up to postoperative 2 hours in both groups. Pain scores in SG and VG were significantly higher up to postoperative 8 hours and 8 am of day 2, respectively, than baseline. Prevalence of rescue analgesia and resentment were not significantly different between groups (SG: 27.3%, VG: 33.3% and SG: 0%, VG: 25%, respectively). Simbadol produced similar analgesic effects to Vetergesic without resentment during drug administration.

## Introduction

Periodontal disease including gingivitis and periodontitis is a plaque-induced pathology and is a serious health problem. It produces pain and inflammation and decrease food intake in both human and companion animals [1–4]. In cats, multiple dental extractions are commonly required for treatment and the procedure can be invasive and painful. Studies in our laboratory showed that cats require long-term analgesic treatment with opioids, local anesthetic blocks and nonsteroidal anti-inflammatory drugs (NSAIDs) after multiple dental extractions [4].

Opioid analgesics are commonly administered as part of perioperative multimodal analgesia for acute pain management in veterinary medicine [5]. However, full agonists of μ-opioid receptors like hydromorphone, oxymorphone and fentanyl are not approved for use in companion animals. Additionally, their unavailability in North America is becoming a critical issue that can jeopardize animal care and welfare.

Buprenorphine is a potent highly lipophilic analgesic opioid that is largely used in the treatment of acute pain. The drug is generally considered as a partial agonist of μ opioid receptors. Buprenorphine is often administered to treat pain in cats as adverse effects have been rarely reported. Cats usually display euphoric behavior and buprenorphine has shown to produce mechanical and thermal antinociceptive effects [6–8]. On the other hand, the drug has failed to provide analgesia in some cats undergoing ovariohysterectomy [9]. For this reason, the drug is commonly administered as part of multimodal analgesia. Indeed, the prevalence of analgesic failure is lower when buprenorphine is administered in combination with other analgesics than alone [7,10,11].

Vetergesic™ (buprenorphine hydrochloride injection, 0.3 mg/mL, Champion Alstoe, Whitby, ON, Canada) is approved for use in cats in several countries. For example in Canada, the labeled dose for intramuscular administration of Vetergesic is 0.02 mg/kg. Indeed, this concentration is similar to formulations of buprenorphine used in humans that are often administered “off-label” in veterinary medicine (e.g. Buprenex™). Simbadol™ (buprenorphine hydrochloride injection, 1.8 mg/mL, Zoetis, Parsippany, New Jersey, USA) is an FDA-approved opioid analgesic for cats. The medication package insert indicates that Simbadol provides 24-hour pain control after a single dose subcutaneously; a total of three injections can be administered for postoperative analgesia. Due to its long-lasting analgesic properties and FDA approval for use in cats, there is an interest in administering buprenorphine for the treatment of pain associated with dental extractions in combination with dental nerve blocks and the administration of NSAIDs in this species. Additionally, it is not known if single or multiple daily injections of Simbadol or Vetergesic using different routes and intervals of administration, respectively, would produce different analgesic effects and frequency of adverse events (i.e. resentment to drug administration). It could be possible that different dosage regimens could still yield similar analgesic effects.

The objective of the study was to evaluate the analgesic efficacy and adverse events of Simbadol in comparison with Vetergesic as part of a multimodal regimen in cats undergoing dental extractions. Our hypothesis was that the two treatments would produce similar postoperative pain scores, adverse events and timing and prevalence of rescue analgesia when using the Glasgow Composite Measure Pain Scale-Feline (CMPS-F) [12].

## Materials and methods

### Study design

The study design was a prospective, blinded, randomized clinical trial. All experimental procedures were approved by the institutional animal care and use committee of the Université de Montréal (18-Rech-1927) and this study is reported according to the CONSORT guidelines [CONSORT guideline; http://www.consort-statement.org]. The experimental study was performed at the Centre hospitalier universitaire vétérinaire (CHUV), the veterinary teaching hospital of the Faculty of Veterinary Medicine of the Université de Montréal, from August 2018 to April 2019.

### Animals

**Fig 1:**
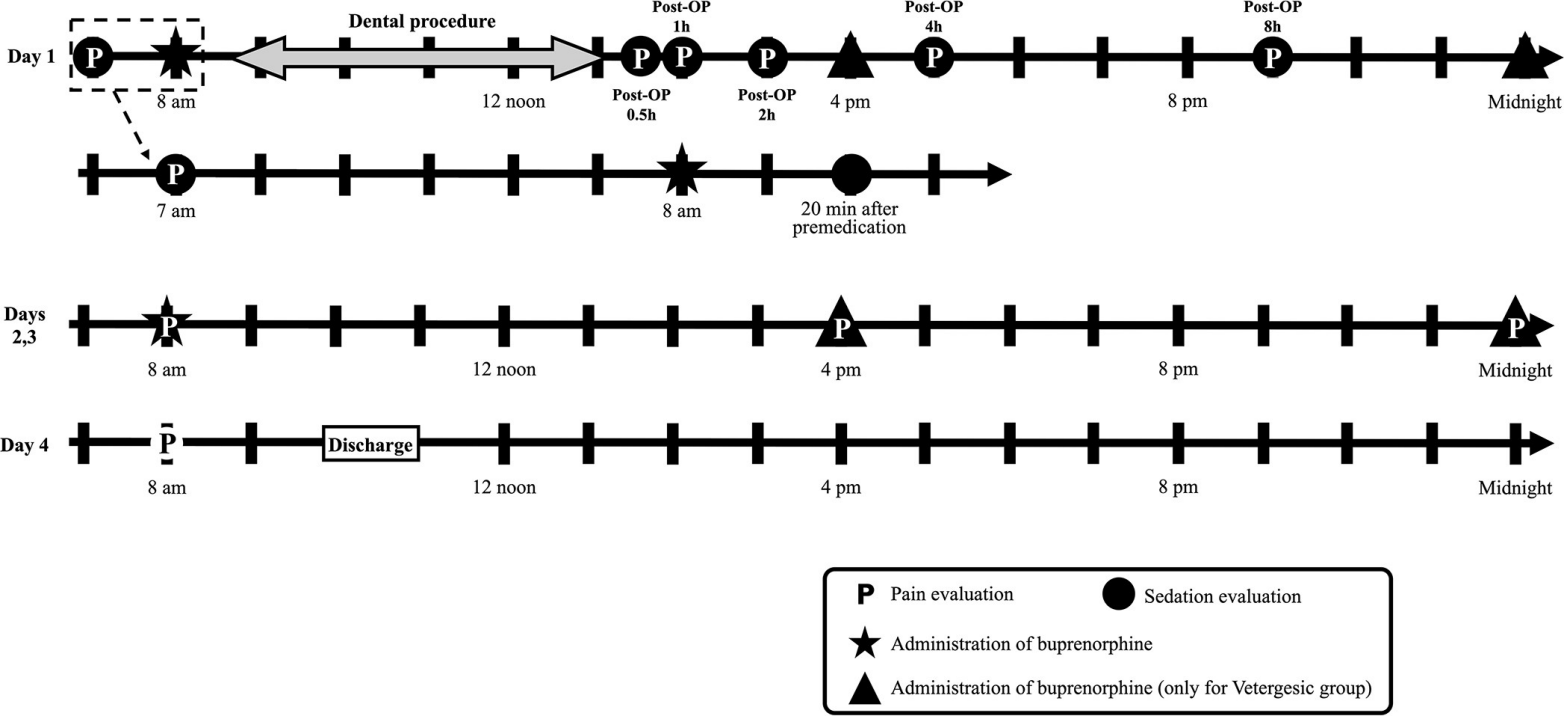
Schematic of time points for administration of buprenorphine during the study. The timeline demonstrates and example of a 4-hour dental procedure in a cat including time points of pain and sedation assessment.

Thirty adult client-owned cats were recruited after informed written consent. Cats were included based on medical records, complete physical examination, and hematology and biochemical panel and had to be free of systemic disease. Cats with body condition score between 3–7 out of 9, and with moderate to severe oral disease were included. Disease severity was determined using a dental scoring system which involved the number and location of teeth extraction: canine tooth: 3 points, third premolar of maxilla or molar of mandible: 2 points, second premolar of maxilla or premolar of mandible: 1 point. A score of 2 points was given if seven or more incisive teeth and/or first premolars of the mandible were extracted; a score of 1 point was given if six or fewer teeth were extracted [4]. The total dental score was calculated and cats with dental score ≥ 6 were included in this study. Cats were excluded if they presented fearful behavior that could impair pain assessment, concurrent medical conditions or diseases (i.e. cancer, renal, cardiovascular, hepatic, or gastrointestinal disease) and/or received any medication including analgesics and antibiotics for up to 10 days before the study had begun. Cats were admitted at day 0 and underwent oral examination, radiographs and treatment under general anesthesia at day 1 (Fig 1). All patients were discharged at day 4.

#### Group allocation

All cats were randomly allocated into one of two treatments groups: Vetergesic group [Vetergesic 0.02 mg/kg intramuscularly (IM) three times a day (8 am, 4 pm and midnight) for 3 days] or Simbadol group [Simbadol 0.24 mg/kg subcutaneously (SC) once a day (8 am) for 3 days]. Randomization was performed using a random permutation generator (http://www.randomization.com) (Fig 1). IM and SC administration were always performed over the epaxial muscles and between the shoulder blades, respectively, by individuals not involved with sedation or pain assessment (ME and PS).

#### Anesthetic and surgical procedures

All cats were premedicated with either Vetergesic or Simbadol at the doses described above. A eutectic mixture of local anesthetic cream (EMLA cream lidocaine 2.5% and procaine 2.5% cream, Astra Zeneca, Mississauga, ON, Canada) was applied and covered with plastic film and adhesive bandage after clipping the hair over the skin of one of the cephalic veins. A 22-G x 1-inch needle intravenous (IV) catheter was aseptically placed in the cephalic vein approximately 20 minutes after premedication. Anesthesia was induced with propofol (Propoflo 28, 10 mg/mL, Zoetis, Kirkland, QC, Canada) administered IV to allow endotracheal intubation after spraying the arytenoid cartilages with 0.05 mL of lidocaine 2% (Lidocaine hydrochloride sterile injection, 20 mg/mL, Vétoquinol N.-A.Inc, Lavaltrie, QC, Canada). The endotracheal tube was then connected to a coaxial Mapleson D system. Anesthesia was maintained with isoflurane vaporized in oxygen by a single veterinarian with experience in anesthesia (ME). Hemoglobin oxygen saturation, heart rate obtained from a lead II electrocardiography, respiratory rate, end-tidal carbon dioxide, inspired and expired concentrations of isoflurane, indirect blood pressure via oscillometry, and rectal temperature were monitored every 5 minutes during anesthesia using a multiparametric monitor (Lifewindow 6000V Veterinary Multiparameter Monitor; Digicare Animal Health, Boynton Beach, FL, USA). Blood pressure was also monitored with a Doppler flow monitor and a sphygmomanometer. The cuff width used for blood pressure monitoring was approximately 40% of the limb circumference. Lactated Ringer’s solution (Lactated Ringer’s Inj. Bag / 500 ml, McCarthy & Sons Service, Calgary, AB, Canada) was administered at 5 mL/kg/hour during the first hour of the procedure. Fluid rates were then adjusted based on the cat’s hydration status and requirements (2–5 mL/kg/hour). If hypotension was observed (mean arterial blood pressure < 60 mmHg), a bolus of the isotonic solution (5 mL/kg over 15 minutes) was given. Dental nerve blocks including the infraorbital, maxillary and/or inferior alveolar mandibular nerve blocks were performed with bupivacaine 0.5% (Sensorcaine, 5 mg/mL, AstraZeneca, ON, Canada) using a 25-G needle based on the location of dental extractions (0.2–0.3 mL/site depending on the number of blocks required after radiographs and approximately 20 minutes before the procedure). The block was repeated if the sympathetic responses to surgical stimulation were observed during dental extractions. The total dose of bupivacaine for all anesthetic blocks did not exceed 2 mg/kg. Meloxicam (0.2 mg/kg, SC, Metacam 5 mg/mL Solution for Injection; Boehringer Ingelheim, Burlington, ON, Canada) was administered at the end of the surgical procedure. Oral administration of meloxicam (0.05 mg/kg, Metacam 0.5 mg/mL Oral Suspension for Cats; Boehringer Ingelheim, Burlington, ON, Canada) were continued for three days at 24, 48 and 72 hours after the first dose according to the label recommendations in Canada. Dental treatment was performed by a resident (JM) and a board-certified veterinarian (YD) of the American Veterinary Dental College (AVDC). Dental parameters [i.e. periodontal disease staging (0–4), gingival, calculus and plaque index (0–2), number of teeth extraction and dental score] were evaluated under general anesthesia as defined by AVDC [AVDC Nomenclature. https://www.avdc.org/Nomenclature/Nomen-Intro.html.]. Anesthesia time (time elapsed from induction of propofol to turning off the vaporizer dial of isoflurane), procedure time (time elapsed from start of dental procedure [i.e. dental scaling] to end of all procedures [i.e. polishing]) and surgery time (time elapsed from the first incision until placement of the last suture) were recorded.

### Sedation scores

Sedation scores were evaluated by an individual (RW) who was unaware of treatment groups using the dynamic and interactive visual analog scale (DIVAS) where 0 was considered as no sedation and 100 as maximum sedation [13]. These evaluations were performed approximately 60 min prior to the premedication (baseline), 20 min after premedication, and at 0.5, 1, 2, 4, 8 hours postoperatively at day 1 (Fig 1).

### Pain scores

The CMPS-F [12] and Feline Grimace Scale (FGS) [14] were used to evaluate pain. Data regarding the FGS are not presented here and will be used as part of additional validation of the tool in cats undergoing dental extractions. The outcome of this study was solely based on the CMPS-F scores. Pain was always assessed by the same individual who also evaluated sedation. Pain scoring was performed at the same time points described above for sedation at day 1 (with the exception of 20 min after premedication), and at 8 am, 4 pm and midnight on days 2 and 3, and at 8 am on day 4 (Fig 1).

### Resentment to drug administration

Resentment was considered any type of escape behavior associated with aversion to drug administration including vocalization, hissing, growling and attempt to bite. Resentment was recorded as present or absent by the individuals who administered buprenorphine during drug administration.

### Rescue analgesia

Cats were administered hydromorphone either at 0.05 mg/kg IV (if the intravenous catheter was in place, at day 1) or 0.1 mg/kg IM (if the intravenous catheter had been removed, at days 2 to 4) if CMPS-F scores were ≥ 5/20. Pain assessment was performed 30 minutes after rescue analgesia to ensure the patient’s comfort. Pain and sedation scores obtained after rescue analgesia were excluded from the statistical analysis, but assessments of sedation and pain were continued until the end of the study. Treatments with buprenorphine were stopped after the administration of hydromorphone.

### Statistical analyses

Statistical analyses were performed using standard statistical software (SPSS Statistics V25, IBM, USA). Power analysis was calculated before the study and indicated that a sample size of 8 cats per group would be required to detect a difference of 3 points between the two groups using the CMPS-F with an alpha value of 0.05, a power of 80% and a standard deviation of 2 points. The sample size was increased to compensate for any individual variability in pain scores and the potential for cats with dental scores ˂ 6 that would lead to patient exclusion.

Data were tested for normality using a Shapiro-Wilk test. Demographic data for each treatment group were compared using independent t-test or Mann-Whitney U test where appropriate. To normalize the distribution of sedation scores, log_10_ transformation was performed after adding one to all values because baseline values were zero. Sedation and pain scores were compared between treatments and between baseline and each time point using a linear mixed model for repeated measures. Time and treatment group, and their interaction were considered as fixed effects. Cat was considered a random effect and dental score was added as a covariate to the model. The best structures of the covariance (first order autoregressive) were assessed using information criteria that measured the relative fit of a competing covariance model. The Benjamini-Hochberg procedure was used to adjust the alpha level for each comparison. The prevalence of rescue analgesia and resentment (dichotomized data) during administration of buprenorphine were compared between treatment groups using Fisher’s exact test. Values of *p* < 0.05 were considered statistically significant.

## Results

Seven cats were excluded from the study; six cats were excluded because of dental scores < 6 and one cat developed fearful behavior during hospitalization after dental treatment. Therefore, 23 cats were included (12 cats in Vetergesic group and 11 cats in Simbadol group). The local anesthetic block was repeated in twelve cats (6 cats in each group). Temporary mild hypotension was observed in twelve cats (6 cats in each group) which improved after the fluid bolus.

One cat in Simbadol group developed upper respiratory disease and conjunctivitis in the evening of day 3. Antibiotics [amoxicillin/clavulanic acid (125 mg/kg/PO BID, Clavamox, Zoetis, Kirkland, QC, Canada) and tetracycline (eye lube TID, Terramycin, Zoetis, Kirkland, QC, Canada)] were administered for 10 days. One cat in Vetergesic group developed asthma and upper respiratory disease at day 2 (i.e. noon) which required antibiotics (amoxicillin/clavulanic acid: 62.5 mg/kg/PO BID for 14 days) and inhalation administration of fluticasone (250 μg/BID, Flovent HFA, GlaxoSmithKline Inc., Mississauga, ON) and salbutamol 100 μg/spray/BID, Ventolin HFA, GlaxoSmithKline Inc., Mississauga, ON). These two cats were discharged without severe clinical signs. Data obtained after the development of clinical signs were excluded from the statistical analysis.

### Demographic data and dental parameters

Breed and gender distribution are shown in Table 1. Demographic data, propofol requirements, and anesthesia, procedure and surgery times are shown in Table 2. Dental parameters are shown in Table 3. There were not significant differences between groups for the information presented in Tables 2 and 3.

**Table 1:** Demographic data including gender, reproductive status and breed of cats undergoing dental extractions and treated with Simbadol or Vetergesic.

	Category	Simbadol (n = 11)	Vetergesic (n = 12)
**Gender**	**Neutered male**	8	5
	**Spayed female**	3	7
**Breed**	**Domestic short hair**	8	8
	**Domestic long hair**	3	4

**Table 2:** Demographic data including age, body weight, body condition score, propofol requirements for anesthetic induction, and anesthesia, procedure and surgery times. Values are expressed as mean ± SD except for body condition score which is reported as median (range).

Variable	Simbadol (n = 11)	Vetergesic (n = 12)	*p* value
**Age (years)**	7.9 ± 2.2	8.5 ± 2.3	0.535
**Body weight (kg)**	5.2 ± 0.8	4.6 ± 0.9	0.154
**Body condition score (1–9)**	5 (5–7)	5 (5–7)	0.260
**Propofol requirements (mg/kg)**	4.9 ± 1.3	5.0 ± 1.1	0.582
**Anesthesia time (min)**	283.6 ± 88.7	313.8 ± 81.0	0.402
**Procedure time (min)**	268.2 ± 89.5	298.3 ± 83.5	0.413
**Surgery time (min)**	210.2 ± 83.7	232.7 ± 86.6	0.534

**Table 3:** Dental parameters including periodontal disease staging, gingival, calculus and plaque index, number of tooth extractions and dental score. Values are expressed as median (range).

Parameter	Simbadol (n = 11)	Vetergesic (n = 12)	*p* value
**Periodontal disease staging (0–4)**	3 (1–4)	3 (1–4)	0.658
**Gingival index (0–3)**	2 (1–3)	2 (1–3)	0.786
**Calculus index (0–3)**	2 (0–3)	2 (1–3)	0.326
**Plaque index (0–3)**	2 (1–3)	2 (1–3)	0.379
**Number of tooth extraction**	11.5 (5–22)	18 (10–23)	0.328
**Dental score (0–28)**	10.5 (8–22)	15.5 (7–25)	0.356

### Sedation scores

DIVAS scores are shown in Table 4. There were no differences between groups (*p* > 0.160, df > 80.20). In both groups, DIVAS scores after sedation and postoperative 0.5, 1 and 2 h were significantly higher than baseline.

**Table 4:** Dynamic and interactive visual analog scale (DIVAS) scores in cats undergoing dental extractions after the administration of Simbadol or Vetergesic. Values are expressed as median (range).

Time points	Groups	DIVAS	*p* value between groups	*p* value compared with baseline
**Baseline**	Simbadol (n = 11)	0 (0)	0.816	
	Vetergesic (n = 12)	0 (0)		
**20 min after premedication**	Simbadol (n = 11)	7 (0–9)	0.453	< 0.0001*
	Vetergesic (n = 12)	6.5 (0–14)		< 0.0001*
**Postoperative 0.5 h**	Simbadol (n = 11)	25 (3–57)	0.160	< 0.0001*
	Vetergesic (n = 12)	37 (17–92)		< 0.0001*
**Postoperative 1 h**	Simbadol (n = 11)	13 (5–41)	0.897	< 0.0001*
	Vetergesic (n = 12)	14.5 (0–86)		< 0.0001*
**Postoperative 2h**	Simbadol (n = 11)	6 (0–36)	0.483	0.0005*
	Vetergesic (n = 12)	2.5 (0–86)		0.0009*
**Postoperative 4 h**	Simbadol (n = 11)	0 (0–26)	0.879	0.097
	Vetergesic (n = 11)	0 (0–74)		0.028
**Postoperative 8 h**	Simbadol (n = 9)	0 (0)	0.807	0.906
	Vetergesic (n = 10)	0 (0–13)		0.502

*Significant difference after adjustment.

### CMPS-F

CMPS-F scores are shown in Table 5. There were no significant differences between groups (*p* > 0.148, df > 44.29). In the Vetergesic group, CMPS-F scores were higher at 4 and 8 hours on day 1 and 8 am on day 2 compared with baseline. In the Simbadol group, CMPS-F scores were higher at postoperative 4 and 8 hours on day 1 compared with baseline (*p* < 0.001 in these time points).

**Table 5:** Pain scores using the Glasgow Composite Measure Pain Scale-Feline (CMPS-F) in cats undergoing dental extractions after the administration of Simbadol or Vetergesic. Values are expressed as mean (SEM).

Time points		Treatments	CMPS-F	*p* value between groups	*p* value compared with baseline
**Day 1**	**Baseline**	Simbadol (n = 11)	0.7 (0.5)	0.858	
		Vetergesic (n = 12)	0.8 (0.4)		
	**Postoperative 0.5 h**	Simbadol (n = 11)	0.9 (0.5)	0.558	0.571
		Vetergesic (n = 12)	0.5 (0.4)		0.438
	**Postoperative 1 h**	Simbadol (n = 11)	1.5 (0.5)	0.148	0.068
		Vetergesic (n = 12)	0.6 (0.4)		0.676
	**Postoperative 2h**	Simbadol (n = 11)	2.0 (0.5)	0.371	0.007
		Vetergesic (n = 12)	1.4 (0.4)		0.126
	**Postoperative 4 h**	Simbadol (n = 11)	2.5 (0.5)	0.920	0.0004*
		Vetergesic (n = 11)	2.4 (0.4)		0.0006*
	**Postoperative 8 h**	Simbadol (n = 9)	2.6 (0.5)	0.759	0.0005*
		Vetergesic (n = 10)	2.8 (0.5)		< 0.0001*
**Day 2**	**8 am**	Simbadol (n = 9)	2.3 (0.5)	0.234	0.004
		Vetergesic (n = 9)	3.1 (0.5)		< 0.0001*
	**4 pm**	Simbadol (n = 8)	1.7 (0.5)	0.775	0.058
		Vetergesic (n = 7)	1.9 (0.5)		0.037
	**Midnight**	Simbadol (n = 8)	1.4 (0.5)	0.883	0.168
		Vetergesic (n = 7)	1.3 (0.5)		0.315
**Day 3**	**8 am**	Simbadol (n = 8)	1.2 (0.5)	0.596	0.372
		Vetergesic (n = 7)	1.5 (0.5)		0.177
	**4 pm**	Simbadol (n = 8)	1.6 (0.5)	0.297	0.080
		Vetergesic (n = 7)	0.9 (0.5)		0.796
	**Midnight**	Simbadol (n = 7)	1.4 (0.5)	0.276	0.208
		Vetergesic (n = 7)	0.6 (0.5)		0.772
**Day 4**	**8 am**	Simbadol (n = 7)	0.6 (0.5)	0.861	0.925
		Vetergesic (n = 7)	0.7 (0.5)		0.953

*Significant difference after adjustment.

### Resentment to drug administration

Resentment was observed during the administration of buprenorphine in three cats in the Vetergesic group (3/12 cats; 25%; two cats at day 2 and one cat at day 3) and none of the cats in the Simbadol group (0/11; 0%) (*p* = 0.12).

### Rescue analgesia

Rescue analgesia was administered to four cats in the Vetergesic group (4/12 cats; 33.3%), and three cats in the Simbadol group (3/11 cats; 27.3%) (Table 6). Prevalence of rescue analgesia was not different between groups (*p* = 0.56).

**Table 6:** Number of cats receiving rescue analgesia at each time point during the study.

Group	Day 1 (postoperative)					Day 2	Days 3 and 4	Number of cats	Frequency of rescue analgesia*	*p* value
	0.5 h	1 h	2 h	4 h	8 h					
**Simbadol** (n = 11)	0	0	0	2	0	1	0	3 (27.3%)	3	0.56
**Vetergesic** (n = 12)	0	0	1	1	1	3	0	4 (33.3%)	6	

*Rescue analgesia could be administered more than once to the same cat.

## Discussion

This study showed that Simbadol produced similar analgesic effects to Vetergesic without resentment during drug administration in cats with oral disease undergoing dental treatment. Pain score were not significantly different between treatments; however, pain scores were significantly increased longer in the Vetergesic group than Simbadol when compared with baseline. This result suggests that the analgesic effects of a single dose of Simbadol (subcutaneous administration of 0.24 mg/kg) could be long-lasting for dental extractions in cats in comparison with the dosage regimens used in the Vetergesic group (intramuscular administration of 0.02 mg/kg every 8 hours). The dose of Vetergesic was based on label recommendations in Canada where the drug is used for postoperative pain relief at 0.01–0.02 mg/kg intramuscularly with an option to repeat a second dose two hours after the first injection, if necessary. Alternatively, the use of other classes of analgesics (i.e. multimodal analgesia) is also recommended in the label as it was done in this study with the combination of local anesthetics and NSAIDs. The frequency of administration for Vetergesic was determined based on the duration of analgesic effect for buprenorphine [15,16]. Additionally, the study attempted to mimic intramuscular injections that would be used in clinical practice in the absence of an intravenous catheter. However, it is reasonable to argue that intravenous administration of buprenorphine could have produced more profound analgesia than the intramuscular route. It is also arguable that the analgesic effects of Vetergesic could have been more appropriate if injections were made every 6 hours, however that would have produced even greater prevalence of resentment during drug administration compromising feline welfare. Indeed, three cats in the Vetergesic group showed resentment and this difference would have been significantly different than Simbadol if one more cat in the Vetergesic group had had resentment. Since intramuscular injections are known to be painful [17], the frequency of injection should be minimized as much as possible for the ethical reasons [18]. The buccal (transmucosal) route of administration could have also been considered in this study. However, it has failed to produce clinical analgesia after administration of buprenorphine in cats [19] especially considering that the cats underwent a dental procedure and the presence of sutures and inflammation could preclude the use of the buccal route. Therefore, finally it was not considered an option for pain relief in this study. In feline practice, the administration of analgesics should be performed based on the patient’s needs using pain scoring systems rather than a predetermined regimen [20]. This is particularly true when considering the individual variability after the administration of intramuscular buprenorphine hydrochloride [6,7]. For example, the duration of thermal antinociception was observed for only 60 minutes even considering a relative long elimination half-life of 460 ± 285 minutes [6]. This gap is often explained by negative hysteresis where plasma concentrations of the drug does not correspond to analgesic efficacy. On the other hand, SC administration of Simbadol 0.24 mg/kg produced thermal antinociception up to 24 hours [8]. This should explain why pain scores returned to baseline values in the morning of day 2 in the Simbadol group. However, both treatments produced similar pain scores and prevalence of rescue analgesia.

There is a possible concern that multimodal analgesia may have biased our results. On the other hand, all cats received meloxicam and local anesthetic blocks with bupivacaine allowing the study design to compare Vetergesic and Simbadol when administered as part of multimodal analgesia. The administration of dental nerve blocks with bupivacaine might have influenced early postoperative pain scores since timing between the last dental nerve block and the end of anesthesia was approximately 1.5 hours. However, in both groups, some cats required early administration of rescue analgesia indicating that buprenorphine in combination with dental nerve blocks and NSAIDs may not provide adequate analgesia in some individuals. These findings were also reported after the administration of hydromorphone in cats undergoing dental extractions highlighting that severe oral disease and dental extractions produce severe pain postoperatively requiring frequent and long-lasting administration of opioids [4]. In this study, an agonist of opioid receptors (hydromorphone) was administered as rescue analgesia in cats pretreated with a partial agonist of μ opioid receptors (buprenorphine). The combination of these two opioid analgesic drugs may be suboptimal and less than ideal. However, pain assessment was continuously performed to ensure patient comfort and to confirm that hydromorphone had been effective.

In this study, cats were included based on the number and location of tooth extraction as previously reported [4]. In the aforementioned study, the severity of oral disease (minimal versus severe) was defined as dental scores ≤ or > 7, respectively, and 91.7% of the cats with severe oral disease required rescue analgesia even after the administration of hydromorphone in the premedication in combination with local anesthetic blocks and NSAIDs. In this study, the cut-off for dental scores was lower (i.e. ≥ 6) than in our previous study because the authors felt that this lower score already produces enough postoperative pain and inflammation allowing to study different analgesic treatments. A lower score also facilitated patient recruitment. However, this could explain the lower prevalence of rescue analgesia in this study (approximately 30%) versus the previous one using hydromorphone in cats. The group allocation was performed randomly, and all demographic data and the dental parameters indicating the severity of oral disease were not different between treatment groups, which would make it reasonable to compare the analgesic efficacy of two treatments.

There are limitations in this study. Firstly, the pain evaluations were performed based on the time points after extubation time and not the administration of buprenorphine in the morning of day 1. Therefore, the patients were evaluated at different time points because of the different duration of surgery. However, anesthetic, procedure and surgical times were not significantly different between groups minimizing this potential bias in pain assessment. Secondly, the doses, concentrations, and routes of administration are different between Vetergesic and Simbadol which may influence their analgesic efficacy in cats. Simbadol is a high-concentration formulation of buprenorphine (1.8 mg/mL) approved for SC administration using high doses of the drug (0.24 mg/kg) whereas Vetergesic presentation has a lower concentration (0.3 mg/mL) and lower recommended doses of administration (0.02 mg/kg IM). It may be arguable that comparisons between the two drugs using such dosage regimens are not appropriate. According to previous studies, Simbadol (0.24 mg/kg SC) and standard concentrations of buprenorphine (0.3 mg/mL; 0.02 mg/kg IM) have different elimination half-life (12.3 hours and 7.7 hours), time to peak plasma concentrations (0.08 hour and 0.05 hours) and duration of antinociceptive effect (24 hours and between 1 and 4 hours when doses of 0.01–0.02 mg/kg are administered), respectively [6,8,20]. Although the route of administration could have been standardized (i.e. subcutaneously), the SC administration of buprenorphine at 0.3 mg/mL did not produce a thermal antinociceptive effect when compared with IM or IV [6]. Thirdly, resentment to drug administration was evaluated using a dichotomized means of assessment (i.e. presence or absence). To the authors’ knowledge, there are no validated means of evaluating resentment to drug administration in cats. Resentment should ideally have been evaluated by an observer who was not aware of the treatment by using a validated scale, if one existed. The resentment to drug administration was likely higher in the Vetergesic group due to the number of injections using the IM route of administration as previously discussed. A more appropriate comparison would involve at least sham/placebo injections three times a day in the Simbadol group, however this was not done to avoid unnecessary added stress to these cats. Finally, pain scores were excluded from statistical analysis after rescue analgesia which could decrease the power of the study and introduce selection bias. However, prevalence of rescue analgesia was used as an important outcome and it was not significantly different between groups corroborating our findings.

## Conclusion

This study showed that both Simbadol and Vetergesic produced similar analgesic effects when using a multimodal analgesic protocol including local anesthetic nerve blocks and meloxicam in cats undergoing dental extractions. However, pain scores in the Vetergesic, but not in the Simbadol group, were still significantly higher in the morning of day 2 when compared with baseline values. This potentially indicates that Simbadol may present longer-sustained analgesic effects than Vetergesic with the dosage regimens used in this study. The frequency and route of drug administration with Vetergesic (i.e. every 8 hours IM) may induce more resentment (i.e. aversive behaviors) than Simbadol (i.e. every 24 hours SC).

## Supporting information

S1 File Raw data. (XLSX)
